# Activating the Expression of Human K-ras^G12D^ Stimulates Oncogenic Transformation in Transgenic Goat Fetal Fibroblast Cells

**DOI:** 10.1371/journal.pone.0090059

**Published:** 2014-03-04

**Authors:** Jianhua Gong, Zhongde Wang, Irina Polejaeva, Ravi Salgia, Chien-Min Kao, Chin-Tu Chen, Guangchun Chen, Liaohai Chen

**Affiliations:** 1 Department of Chemistry and Biochemistry, Utah State University, Logan, Utah, United States of America; 2 Department of Animal, Dairy, and Veterinary Sciences, Utah State University, Logan, Utah, United States of America; 3 Department of Radiology, University of Chicago, Chicago, Illinois, United States of America; Northwestern University Feinberg School of Medicine, United States of America

## Abstract

Humane use of preclinical large animal cancer models plays a critical role in understanding cancer biology and developing therapeutic treatments. Among the large animal candidates, goats have great potentials as sustainable sources for large animal cancer model development. Goats are easier to handle and cheaper to raise. The genome of the goats has been sequenced recently. It has been known that goats develop skin, adrenal cortex, breast and other types of cancers. Technically, goats are subject to somatic cell nuclear transfer more efficiently and exhibit better viability through the cloning process. Towards the development of a goat cancer model, we created a transgenic goat fetal fibroblast (GFF) cell as the donor cell for SCNT. Human mutated K-ras (hK-ras^G12D^) was chosen as the transgene, as it is present in 20% of cancers. Both hK-ras^G12D^ and a herpes simplex viral thymidine kinase (HSV1-tk) reporter genes, flanked by a pair of LoxP sites, were knocked in the GFF endogenous K-ras locus through homologous recombination. Following Cre-mediated activation (with a 95% activation efficiency), hK-ras^G12D^ and HSV1-tk were expressed in the transgenic GFF cells, evidently through the presence of corresponding mRNAs, and confirmed by HSV1-tk protein function assay. The hK-ras^G12D^ expressing GFF cells exhibited enhanced proliferation rates and an anchorage-independent growth behavior. They were able to initiate tumor growth in athymic nude mice. In conclusion, after activating hK-ras^G12D^ gene expression, hK-ras^G12D^ transgenic GFF cells were transformed into tumorgenesis cells. Transgenic goats via SCNT using the above-motioned cells as the donor cells have been established.

## Introduction

Mice are the most commonly used animal model due to the vast array of reagents and gene manipulation strategies currently available for this species. However, the small size of the mouse and its anatomical structures present problematic issues when measuring the pathophysiologic parameters of cancer or other diseases. This is especially evident when comparing the vastly different physiologic values between mice and humans. The use of large animals for modeling cancer would overcome these issues. Large animals (dogs, sheep, goats, pigs, and nonhuman primates) have large organs and blood volumes that allow repeated blood sampling, which can provide critical data for kinetic pharmacologic studies, a mission impossible in small animal studies. The results obtained from large animal experiments can be applied easily to human protocols. Given the state-of-the-art vivarium infrastructure for large animals and strong efforts in large animal cloning at Utah State University (USU), we are carrying out an initiative to develop large animal cancer models using transgenic goats. The choice of goats for cancer model development is motivated by 1) goats develop skin, adrenal cortex, breast and other types of cancers [1]–[4]; 2) goat genome has been sequenced recently [5]; and 3) comparing to pigs or other large animals, goats are more robust to the cloning process, easier to handle and less expensive to raise, which makes them sustainable as an animal model source.

K-ras, a 21 kDa membrane-bound GTPase, which regulates cell growth, proliferation, and differentiation, is a key player in the development and in carcinogenic processes and tumor maintenance [6]–[8]. K-ras mutations can prevent the enzyme from hydrolyzing GTP, resulting in an up-regulation of downstream signaling activity, which leads to uncontrolled proliferation, resistance to apoptosis, metastasis, and ultimately cancer [9], [10]. It has been well documented that K-ras genes are mutated in ∼20% human cancers, ranging from pancreatic cancer, lung cancer, and breast cancer, among others [11]. Accordingly, we chose mutated human K-ras as the transgene to create a mutated human K-ras transgenic goat. The transformation of the mutated human K-ras transgenic goat to a K-ras transgenic goat cancer model is inspired by the reports that expression of K-ras in transgenic mice induces tumors [12]–[14]. We hypothesized that the expression of mutated K-ras gene should also result in tumor growth in goats. In an unlikely event that the expression of mutated K-ras would not lead to the tumor phenotypes in goats, the availability of a transgenic goat with inducible expression of oncogenic human K-ras would still be a great asset for studying the mechanism of K-ras related bio-processes and for screening and testing potential drugs related to K-ras.

More specifically, we aimed at developing a K-ras transgenic goat model for lung cancer. Lung cancer is the most lethal cancer, accounting for almost one third of total cancer mortality. A high percentage of lung cancers express K-ras gene with mutations in codon 9–13 [16]. The site of K-ras mutation is confined almost entirely to codon 12, and the spectrum of mutations is limited such that valine, arginine, aspartic acid, or cysteine is substituted for the normally encoded glycine [17]. This region in K-ras is highly conserved and is identical among mouse, goat, and human.

Our approach for developing a K-ras transgenic large animal model uses somatic cell nuclear transfer (SCNT) method to produce genetically modified goats expressing inducible (through LoxP/Cre recombination) human mutated K-ras oncogene (hK-ras^G12D^ thereafter) and a herpes simplex viral thymidine kinase HSV1-tk gene, a well-established reporter gene. HSV1-tk, although in the presence of goat endogenous tk, can be specifically detected by isotope labeled thymidine analog using positron emission tomography (PET) imaging; therefore, the expression of the hK-ras^G12D^ gene upon Cre-mediated activation can also be detected [18], [19]. The incorporation of a reporter gene to the hK-ras gene has profound effect on producing a transgenic large animal with a latent transgenic disease-caused gene(s) embedded in its genome. Large animals have a much longer lifespan, and the effect of activated transgene(s), as well as the appearance of disease phenotype, could take a long time to manifest. Capabilities of knowing when and where the transgene product(s) is expressed, as well as whether there are any pre-symptom signatures due to the function of transgene product(s) will shed light on the developmental nature (disease initiation) of model development. The reporter gene will enable imaging the effectiveness of hK-ras activation and hK-ras location/expression level upon activation in transgenic goats by PET.

In this report, we focus on *in vitro* studies related to the development of a hK-ras transgenic goat, including the vector construction, development of transgenic cells, *in vitro* Cre-mediated activation of hK-ras^G12D^ and HSV1-tk gene expression, and phenotyping of the corresponding activated cells. Guided through the genome sequences of goat [5], we have knocked in both hK-ras^G12D^ and HSV1-tk gene under the same upstream promoter in the goat endogenous K-ras locus through homologous recombination. Transgenic cells were obtained, and the Cre-mediated activation of hK-ras^G12D^ stimulated oncogenic transformation in goat fetal fibroblast (GFF) cells. To the best of our knowledge, this study is the first to report the transformation of goat fibroblast to tumor genesis under the influence human mutated K-ras gene.

## Methods

### Reagents

Restriction enzymes (FastDigest Xho I, HindIII, AfIII, BglII, AgeI, NotI and BamHI) were purchased from Thermo Fisher Scientific Inc. *Taq* DNA polymerase was obtained from TaKaRa (Cat: KA4301BA). High-fidelity DNA polymerase was from Bio-Rad (Cat: 172-5330) and the In-Fusion HD Cloning Kit was acquired from Clontech (Cat: 639645). The primers used in this study were designed according to the corresponding genes listed (See Table S1 in File S1) and synthesized by Integrated DNA Technologies Inc. DNA sequencing was performed by Center for Integrated BioSystems (CIB) at Utah State University. Electroporation devices of Amaxa™ 4D-nucleofector (Lonza) and P3 Primary Cell 4D-Nucleofector™ X Kit (Lonza) were used in this study. Goat genomic DNA was extracted using PureLink Genocmic kit (Invitrogen Cat: K1820).

### Construction of targeting vector: pKO2.1-LSL-hK-ras^G12D^-IRES-HSV1-tk

Goat genomic DNA was extracted from GFF. The DNA fragments used as homology arms were a 2 kb goat DNA fragment derived from the 400 bp upstream of K-ras exon 1 and a 7 kb fragment from the downstream of K-ras exon 1. The cDNA of human K-ras^G12D^ was amplified from pcDNA3-kras^G12D^ (a gift of Dr. Patrizio Castagnola, National Cancer Research Center, Genova, Italy) and then subcloned into pLOX-gfp-iresTK (Addgene plasmid 12243). LSL (LoxP-Stop-LxoP) fragment was amplified from PGKneotpAlox2 (Addgene plasmid 13444). The 7-kb of the long homology arm was first inserted into pKO2.1 (Addgene plasmid 22674). Next, in-fusion cloning procedure was performed to insert 3 directional cloning PCR amplicons (short homology arm, LSL fragment and hK-ras^G12D^-IRES-HSV1-tk) into linearized pKO2.1 in a single reaction. The correct recombinant colonies were screened by colony PCR and then verified by the digestion analysis of restriction endonuclease. The junction sequence of targeting vector: pKO2.1-LSL-hK-ras^G12D^-IRES-HSV1-tk was confirmed by sequencing.

### Transfection and detection of targeted cell clones

The primary goat fetal fibroblast cells were isolated from 40-d goat fetus as previously described [20] and then were used for the transfection at passage 2 or 3. The linearized targeting vector: pKO2.1-LSL-hK-ras^G12D^-IRES-HSV1-tk/AgeI was electroporated into GFF. Fibroblast colonies resistant to neomycin were expanded and then genotyped by PCR to detect the presence of the transgene and homologous recombination events.

### Removal of stop cassette by Cre

Removal of the Stop element from the LSL-hK-ras^G12D^-IRES-HSV1-tk allele was achieved by the use of Adenovirus-Cre (University of Iowa, Gene Transfer Vector Core) and then verified by PCR.

### MTT assay

Cells were seeded in 96-well plate and the evaluation of cell growth using the MTT assay every other days.

### Ganciclovir Sensitivity Assay

Cells were seeded in 96-well plates (2000 cells/well) and were grown for 24 h. The cell culture medium was replaced daily with ganciclovir-containing medium (Biotang Inc.) at different ganciclovir concentrations. MTT assay was used to measure cell viability 5 days later.

### Clonogenic Assay

Five hundred cells were seeded into each well of 6-well plate in duplicate, and colonies were stained 7–10 days later with 0.2% crystal violet in 80% methanol.

### Anchorage-Independent Growth Assay

For analysis of anchorage-independent growth, cells were trypsinized and 10,000 cells per well of 6-well plate were seeded in medium containing 1.6% Methyl cellulose (Sigma, M0512) with 10% fetal calf serum and plated over a layer of 0.9% agar-coated six-well plates. Standard medium (1 mL) was added to the top of the gelled matrix and colonies were grown for 21 days. After 21 days in culture, colonies were counted in five random three-dimensional fields per well and photographed.

### RT-PCR and restriction fragment length polymorphism analysis

Total RNA was extracted from cells using TRIzol® (Invitrogen). cDNA was synthesized using 2 µg of total RNA with the SuperScript II First-Strand Synthesis using oligo (dT) primer System (Invitrogen). Aliquots of the reaction mixture were used for the subsequent PCR amplification. The primer of HSV1-tk: 5′- CAGCAAGAAGCCACGGAAGT-3′ (sense) and 5′- GGCCCGAAACAGGGTAAATAA-3′ (antisense). To detect transcripts of mutant KRAS, PCR–restriction fragment length polymorphism (RFLP) method was done as described [21]. The primer sequences for K-ras amplification were 5′-GACTGAATATAAACTTGTGGTAGTTGGACCT-3′ (sense) and 5′-TCCTCTTGACCTGCTGTGTCG-3′ (antisense). The sense primer was designed to introduce a base substitution that created a BstNI recognition site for the WT codon 12 (GGT), but not for the codon 12 with the K-ras mutation. PCR products (15 µL) were digested with 30 units of BstNI (New England Biolabs) at 60°C for 3 hours, and were run on 3% agarose gels stained with ethidium bromide.

### Telomerase assays

Telomerase assay was measured with a telomerase ELISA kit (Roche) according the manufacturer's instructions.

### *In vivo* xenograft growth and ^18^F-FDG PET imaging

The protocols that involved laboratory animals were performed in accordance with the recommendations in the Guide for the Care and Use of Laboratory Animals of National Institutes of Health and were approved by the Institutional Animal Care and Use Committee of Utah State University (IACUC #: 2118). All surgery was performed under isoflurane anesthesia, and all efforts were made to minimize suffering.

*In vivo* tumor growth was examined by injecting cells into six week-old female Nu/J (JAX) mice. A total 5×10^6^ cells were injected into nude mice subcutaneously. Tumor sizes were followed after 2 weeks and were measured for 11 weeks.

^18^F-FDG was purchased from the Huntsman Cancer in Utah University, and reconstituted with sterile saline. The mice were fasted for 12 h before ^18^F-FDG injection. The animals were administered 200 µCi 18F-FDG via tail vein injection under isoflurane anesthesia. Animals remained conscious and were allowed free access to water during a 2 h uptake period. The mice were anesthetized immediately before scanning and were imaged in the prone position for 20 min. PET scans and image reconstruction were performed using a panel PET scanner developed by University of Chicago.

## Results

### Construction of targeting vector and generation of transgenic cells

**Figure 1:**
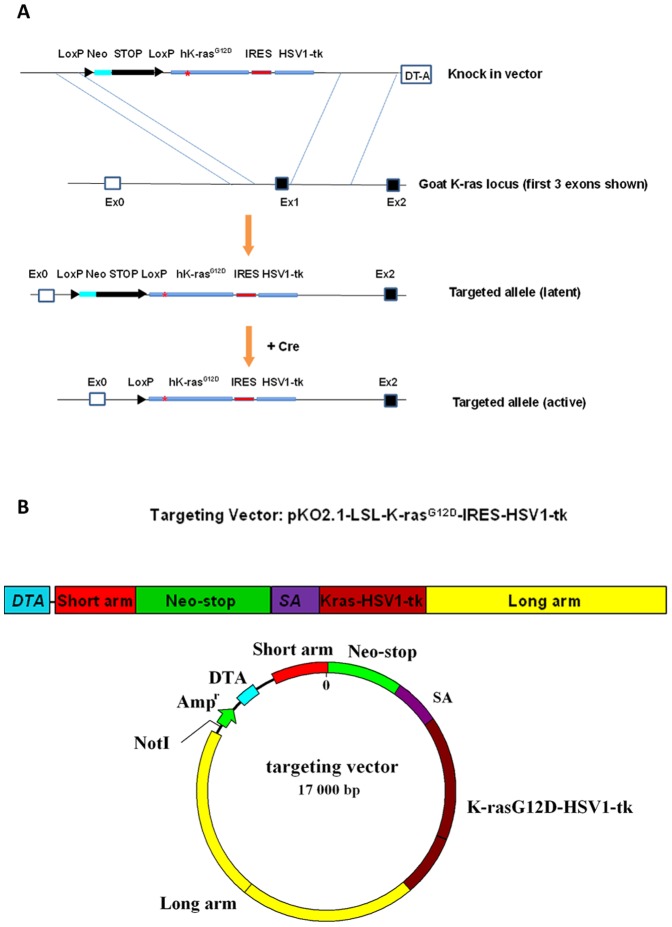
Schematic representation of the conditional hK-Ras^G12D^ construct. A, Targeting strategy of knock-in of the hK-ras^G12D^ to goat K-ras exon 1 locus and its activation by Cre; B, Schematic outline of the cloning strategy for the construction of targeting vector: pKO2.1-LSL-hK-ras^G12D^-IRES-HSV1-tk.

Figure 1A depicted the overall strategy for knock-in of hK-ras^G12D^ oncogene and HSV1-tk reporter gene in goat fibroblast cells and the corresponding conditional activation. Both genes were introduced to the exon 1 locus of goat endogenous K-ras gene. The hK-ras^G12D^ was transcribed together with the HSV1-tk due to their linkage through the internal ribosomal entry site (IRES). The transcription of hK-ras^G12D^ and HSV1-tk genes was inhibited by a transcriptional termination sequence (LoxP-Stop-LoxP, LSL). Both hK-ras^G12D^ and HSV1-tk were expressed under the control of goat K-ras promoter and activated by transfection of Cre gene following the deletion of LoxP-neomycin-stop-LoxP cassette. Figure 1B illustrates a schematic outline for the construction of targeting vector pKO2.1-LSL-hK-ras^G12D^-IRES-HSV1-tk.

**Figure 2:**
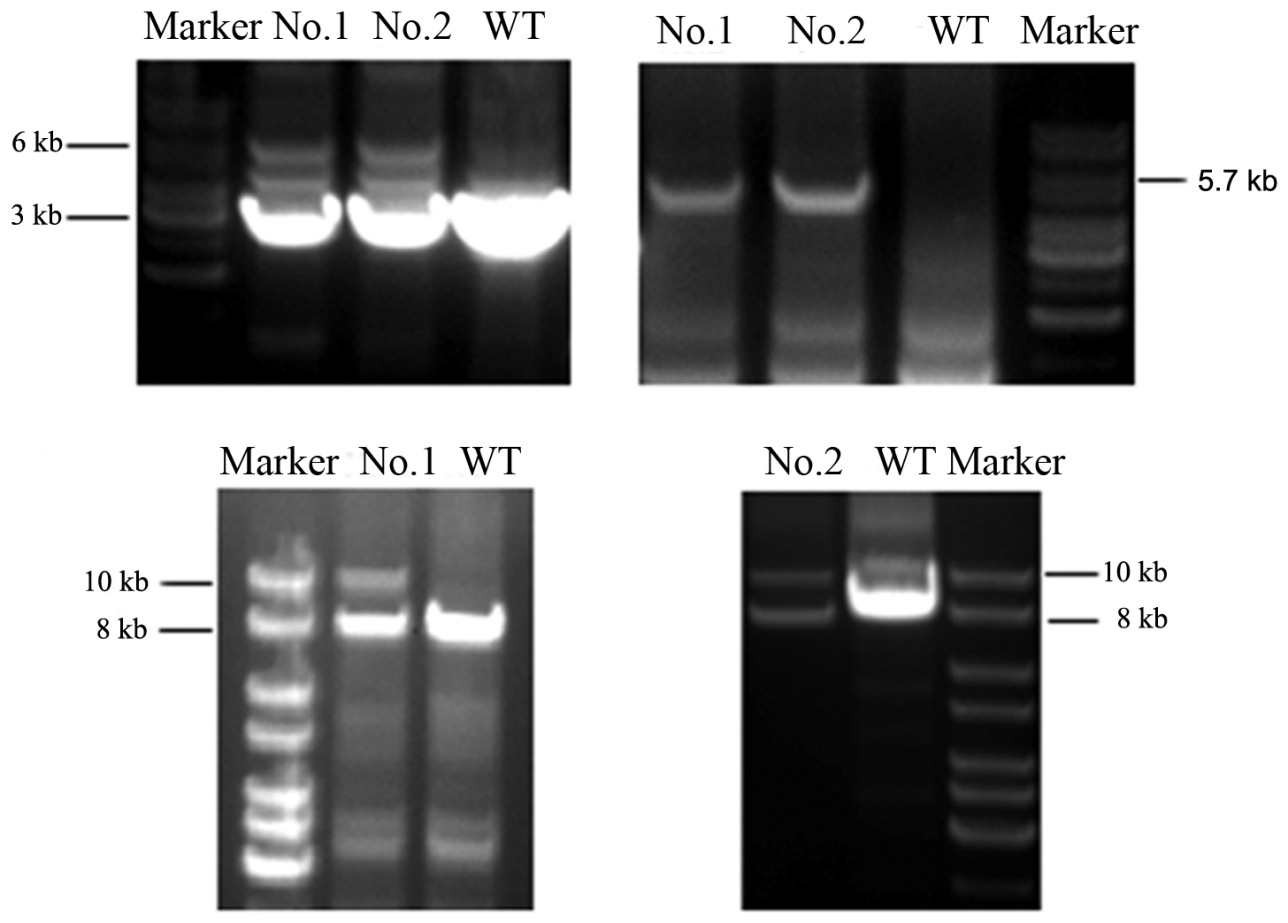
Results of screening PCR in two targeting GFF cells. A, PCR was performed using primers V01 and V02. The wild type allele was denoted by the 3-hK-ras^G12D^-IRES-HSV1-tk allele was denoted by 6 kb fragment. B, PCR was performed using the primers V01 and V03. The wild type allele could not be denoted by any fragment using these pair of primers, and only the recombined LSL-hK-ras^G12D^-IRES-HSV1-tk allele was denoted by 5.7 kb fragment. The fragments in lane1 and lane2 were purified from the gel and then were subject to sequencing. C, PCR was performed using primers V04 and V05. The wild allele type was denoted by the 8 kb fragment, and the recombined LSL-hK-ras^G12D^-IRES-HSV1-tk allele was denoted by 10 kb fragment. WT: wild-type GFF cells. No. 1 and No. 2: Targeting cells. Position and orientation of PCR primers used for the analysis are depicted.

Details of the vector construction are provided in the supplementary material (See Figure S1–S5 in File S1). In brief, to generate the targeting construct of pKO2.1-LSL-hK-ras^G12D^-IRES-HSV1-tk, cloning and long-rang PCR were used to amplify a large genomic region spanning the exons. In-fusion PCR was used to ligate 3 inserted fragments into the targeting vector in a one-step reaction. The essential elements of targeting vector, including the transcriptional termination LSL, hK-ras^G12D^-IRES-HSV1-tk and all the junction of ligation, were sequenced, and the results showed correct sequences with no mutations or reading frame shift. Subsequently, the knock-in vector was linearized and electroporated into GFF cells. Fibroblast colonies resistant to neomycin (600 µg/ml) were expanded and 56 neo-resistant colonies were obtained. To screen for homologous recombination by PCR, three pairs of primers were designed and used to identify the targeted locus (See Figure S6 in File S1). PCR-based screening revealed that 2 out of 56 analyzed GFF colonies contained correct knock-in targeting gene (Figure 2). Two correct targeting cell lines (No. 1 and No. 2), in which the homologous recombination event occurred, were established.

### Activating hK-ras expression in LSL-hK-ras^G12D^-IRES-HSV1-tk transgenic GFF by Cre-LoxP recombination

The activation of hK-ras^G12D^ expression was carried out by infecting the cells with high doses of Adenovirus-Cre-GFP to remove the transcription termination cassette LSL. The Cre recombinase caused the excision of the transcriptional Stop element; thus, resulting in hK-ras^G12D^ expression.

**Figure 3:**
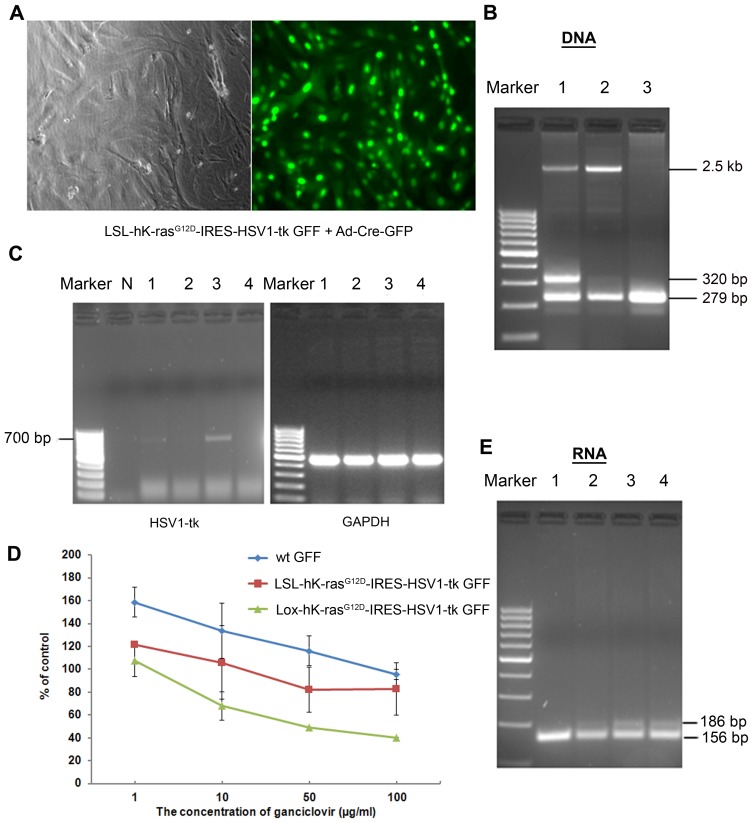
Activating hK-ras expression in LSL-hK-ras^G12D^-IRES-HSV1-tk GFF cells by Adenovirus -Cre-GFP. A. Morphology of GFP positive cells 48-Cre-GFP. Phase contrast (Left panel) and florescent micrographs (Right panel) were shown at 100× magnification. B. Efficient excision of the LSL cassette in the cell culture. PCR analysis of genomic DNA prepared from the different GFF cells. The wild type allele was devoted the 279 bp fragment. The LSL-hKras^G12D^-IRES-HSV1-TK allele was devoted 2.5 kb fragment. The Lox- hKras^G12D^-IRES-HSV1-TK allele was devoted the 320 bp fragment. 1: Lox- hK-ras^G12D^-IRES-HSV1-tk GFF cells, 2: LSL-hK-ras^G12D^-IRES-HSV1-tk GFF cells, 3: wild-type GFF cells. C. mRNA of HSV1-tk in GFF cells. Marker: 100 bp ladder, 1: LSL-hK-ras^G12D^-IRES-HSV1-tk GFF was infected with Adenovirus-Cre-GFP 48 h. 2: LSL-hK-ras^G12D^-IRES-HSV1-tk GFF cells. 3: Lox- hK-ras^G12D^-IRES-HSV1-tk GFF cells, 4: wild-type GFF cells, N: negative control (H_2_O as template). D. Dependence of ganciclovir concentrations against GFF cell viability using MTT assays. The results were expressed as the percentage of living cells in treated conditions at various concentrations of ganciclovir with respect to ganciclovir-free cultures. E. Specific reduction of mutant hK-ras transcripts in GFF cells. BstNI digestion cuts the WT K-ras allele to produce a 156-bp DNA fragment, whereas the mutant hK-ras allele remains uncut to produce a 186-bp DNA fragment. 1: wild-type GFF cells, 2: LSL-hK-ras^G12D^-IRES-HSV1-tk GFF cells, 3: Lox- hK-ras^G12D^-IRES-HSV1-tk GFF cells, 4: LSL-hK-ras^G12D^-IRES-HSV1-tk 48 h post infection.

Forty-eight hours after the infection of LSL-hK-ras^G12D^-IRES-HSV1-tk GFF cells with recombinant adenovirus encoding Cre recombinase, the fluorescence of GFP positive cells could be observed under the fluorescence microscope (Figure 3A). By comparing both bright field and florescence images, greater than 95% infected efficiency was achieved. The infected cells were cultured continuously for 4 weeks until the dominant colonies were appeared, which was named as Lox-hK-ras^G12D^-IRES-HSV1-tk GFF cells.

The removal of the stop element and neomycin gene after infection was confirmed by PCR using genomic DNA extracted from GFF cells. PCR primers are illustrated (see Figure S7 in File S1). Only 279 bp fragment was amplified in wild type GFF cells and both 279 bp and 2.5 kb fragments were amplified in LSL-hK-ras^G12D^-IRES-HSV1-TK GFF cells. Only DNA prepared from the Lox-hK-ras^G12D^-IRES-HSV1-TK GFF cells gave a product, in which the molecule weight was 40 bases larger than that from the wild-type allele generated from the hK-ras^G12D^ allele containing a single loxP site (Figure 3B). The co-existence of 2.5 kb fragment band could be due to the incomplete excision by Cre recombinase.

Since the goat endogenous K-ras locus was targeted in the LSL-hK-ras^G12D^-IRES-HSV1-tk GFF cells, endogenous levels of hK-ras^G12D^ and HSV1-tk proteins should be expressed after the removal of the Stop element. Figure 3C is the result of examination of HSV1-tk mRNA by RT-PCR. The result showed that the HSV1-tk was expressed in Lox-hK-ras^G12D^-IRES-HSV1-tk GFF cells as well as in the LSL-hK-ras^G12D^-IRES-HSV1-tk GFF cells infected with Adenovirus-Cre-GFP for 48 h (Figure 3C). It thus proved that the induced hK-ras allele was efficiently recombined and expressed.

The function of the HSV1-tk transgene in activated Lox-hK-ras^G12D^-IRES-HSV1-tk GFFs was determined by inhibition of cell proliferation when the cells were incubated with Ganciclovir (GCV), a specific substrate of HSV1-tk. Three groups of GFF cells were treated with GCV at increasing concentrations (from 1 to 100 µg/ml), followed by incubation for 5 days. The cytotoxicity of the cells was assayed in triplicate for each concentration using MTT assays. As shown in Figure 3D, Lox-hK-ras^G12D^-IRES-HSV1-tk GFF cells were very sensitive to the GCV and their growth was significantly inhibited at GCV concentration of 100 µg/ml. At the same time, cytotoxicity towards GCV concentrations in LSL-hK-ras^G12D^-IRES-HSV1-tk GFFs and wild-type GFF cells remained unaffected even at 100 µg/ml of GCV.

Due to the lack of commercial antibody against mutant hK-ras, we used PCR-restriction fragment length polymorphism (RFLP) analysis to verify the existence of mutant hK-ras in the genome. In both transgenic Lox-hK-ras^G12D^-IRES-HSV1-tk GFF and LSL-hK-ras^G12D^-IRES-HSV1-tk GFFs cells infected by Adenovirus-Cre-GFP for 48 h, PCR-PFLP analysis indicated the presence of 186-bp band associated with mutant hK-ras in transgenic cells. On the contrary, wild type GFF cells only had 156-bp cDNA fragment (Figure 3E). The presence of dual 186-bp mutant band and 156-bp cDNA band in transgenic Lox-hK-ras^G12D^-IRES-HSV1-tk GFF cells suggested that two types of K-ras existed in two alleles, hK-ras^G12D^ and goat endogenous K-ras.

### Phenotyping transgenic Lox-hK-ras^G12D^-IRES-HSV1-tk GFF cells

**Figure 4:**
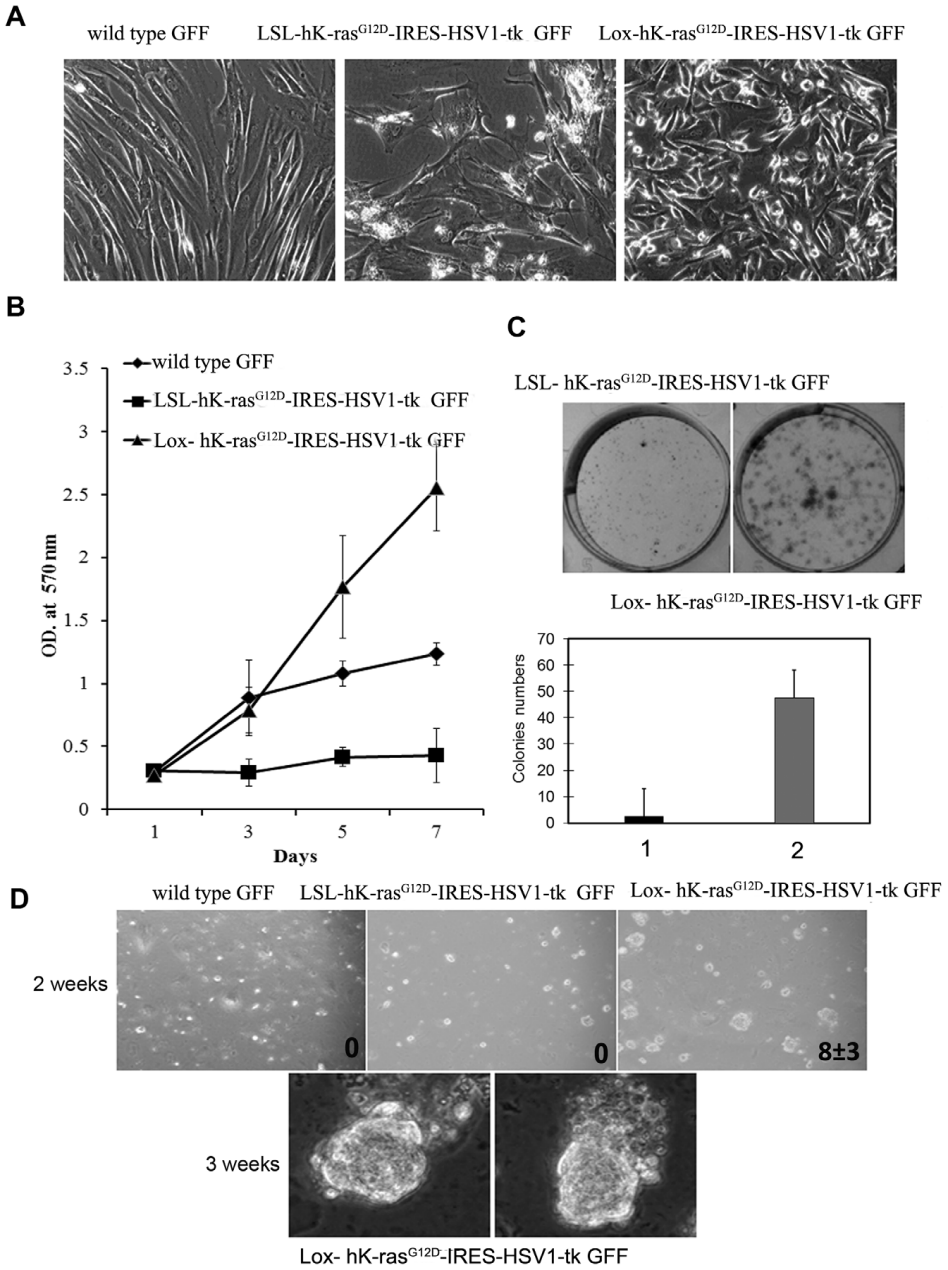
Phenotypic changes of transformed cells. A. Morphological properties of transgenic GFF cells. Phase contrast micrographs were shown at 200× magnification. B. Proliferation curves of wild-type GFF cells, LSL-hK-ras^G12D^-IRES-HSV1-tk and Lox-hK-ras^G12D^-IRES-HSV1-tk GFF cells to indicate enhanced proliferation. 2000 cells per well were seeded in the 96-well plate and incubated for indicated time. The cells were determined by MTT assay. C. Quantification of colony formation between LSL-hK-ras^G12D^-IRES-HSV1-tk and Lox-hK-ras^G12D^-IRES-HSV1-tk GFF cells. 1, LSL-hK-ras^G12D^-IRES-HSV1-tk GFF cells. 2, Lox-hK-ras^G12D^-IRES-HSV1-tk GFF cells. Columns, average of five random fields per dish from two dishes; bar, standard deviation (*, p<0.01; chi-square test). D. The anchorage-independent growth of transgenic cells. Cells were suspended in methylcellulose to evaluate anchorage-independent growth potential over 21 days. Bright field micrographs were shown at 100× magnification (2 weeks) and 200× magnification (3 weeks).

The morphology of Lox-hK-ras^G12D^-IRES-HSV1-tk GFF cells was quite different from the appearance of classic fibroblast (Figure 4A). It was observed that LSL-hK-ras^G12D^-IRES-HSV1-tk GFF cells could grow very quickly at an early passage, but began to grow very slowly after passage 5 and became senescent [22]. Senescent LSL-hK-ras^G12D^-IRES-HSV1-tk GFF cells were unable to divide and exhibited an enlarged and flattened morphology (Figure 4A). On the contrary, Lox-hK-ras^G12D^-IRES-HSV1-tk GFF cells did not undergo the senescent stage and kept fast proliferation. As shown in Figure 4B, transgenic human K-ras^G12D^-expressing fibroblasts featured an enhanced proliferative property. Lox-hK-ras^G12D^-IRES-HSV1-tk GFF cells had a doubling time of 24 hours, obtained from the growth curve and calculated by the formula of log (final number of collected cells/initial number of seeded cells)/log2.

Lox-hK-ras^G12D^-IRES-HSV1-tk GFF cells were further characterized using colony formation assay to determine the consequences of hK-ras^G12D^ expression. We observed that the activation of hK-ras^G12D^ expression enabled an anchorage-independent growth. Whereas the wild type GFF cells and LSL-hK-ras^G12D^-IRES-HSV1-tk GFF cells did not form any colonies in methylcellulose, Lox-hK-ras^G12D^-IRES-HSV1-tk GFF cells showed spherical colony formation with 2 weeks of seeding, and expanded colonies were observed within 3 weeks (Figure 4D). The anchorage-independent growth behavior associated with the Lox-hK-ras^G12D^-IRES-HSV1-tk GFF cells suggested the occurrence of neoplastic transformation after activating K-ras expression and indicated that Lox-hK-ras^G12D^-IRES-HSV1-tk GFF cells are transformed cells.

Unexpectedly, no telomerase activities were observed in Lox-hK-ras^G12D^-IRES-HSV1-tk GFF cells, wild type GFF cells and LSL-hK-ras^G12D^-IRES-HSV1-TK GFF cells (Data not show here). The Lox-hK-ras^G12D^-IRES-HSV1-tk GFF cells at passage 20 were still telomerase-negative. Similar observations that activation of telomere maintenance strategies is not necessary an obligated characteristic feature of tumorigenic cells were also obtained in the literature [23].

### Tumorigenesis in athymic nude mice

**Figure 5:**
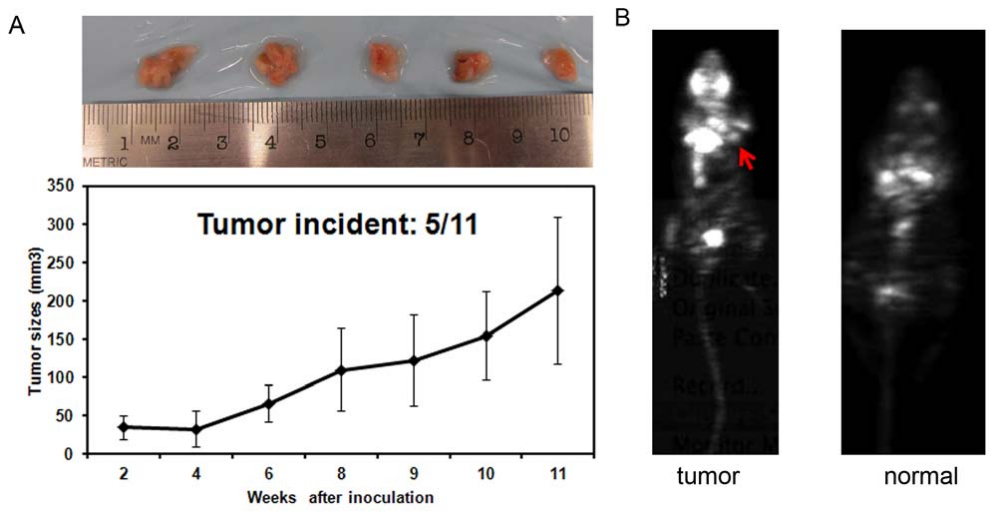
Tumor formation in mice inoculated with Lox-hK-ras^G12D^-IRES-HSV1-tk GFF cells. A. Tumor growth curves of mice bearing Lox-hK-ras^G12D^-IRES-HSV1-tk GFF cells. Inset. Dissected tumor masses from the 5 mice. B. Coronal microPET images of nude mouse inoculated with Lox-hK-ras^G12D^-IRES-HSV1-tk GFF cells after injection of ^18^F-FDG. Red arrow indicates palpable tumor mass. Mouse inoculated with LSL-hK-ras^G12D^-IRES-HSV1-tk GFF cells was used as a control.

To further confirm the capability of tumorigenesis *in vivo* after activating hK-ras^G12D^ expression, both LSL-hK-ras^G12D^-IRES-HSV1-tk and Lox-hK-ras^G12D^-IRES-HSV1-tk GFF cells were introduced via s.c. transplantation into athymic nude mice. We monitored tumor formation and tumor growth for 11 weeks post cell injection (Figure 5A). Mice receiving LSL-hK-ras^G12D^-IRES-HSV1-tk GFF cells did not exhibit any tumor growth during the course of 11 weeks, while mice inoculated with Lox-hK-ras^G12D^-IRES-HSV1-tk GFF cells resulted in palpable tumors within 2 weeks, with tumor formation incidence of 40% during 11 weeks (2/5).

To further confirm the tumor mass, Positron Emission Tomography (PET) was deployed to image the tumor mass that developed in the mice using 2-deoxy-2-(18F)-fluoro-D-glucose (^18^F-FDG) probe. 18F-FDG, as a glucose analog, was taken up by high-glucose-consuming tumor cells, which yielded a higher contrast associated with tumor mass in PET image. As shown in Figure 5B, palpable mass exhibited enhanced ^18^F-FDG uptake in the mice inoculated with Lox-hK-ras^G12D^-IRES-HSV1-tk GFF cells when compared with the control group comprising the mice inoculated with the non-activated transgenic cells.

In conclusion, after activating hK-ras^G12D^ gene expression, hK-ras^G12D^ transgenic GFF cells were transformed into tumorgenesis cells.

## Discussion

In this work, we developed transgenic goat cells with hK-ras and HSV1-tk genes using primary GFF cells. Comparing with similar works on mouse model development [12]–[15], we found wild-type GFF cells are quite different from mouse embryonic fibroblast (MEF) cells. Wild-type MEF cells senesced after 5–10 passages, while wild-type GFF cells grew very fast and kept proliferating after 20 passages. Once wild-type GFF cells were turned into transgenic LSL-hK-ras^G12D^-IRES-HSV1-tk cells, they began to grow slowly after 5 passages and became senescent, while there was not much difference in growth rate between wild-type MEF and transgenic MEF cells. After hK-ras was activated in transgenic GFF cells, the activated cells started to grow aggressively — faster than the wild-type GFF and became tumor genesis (Figure 4). A relatively faster growth rate is a characteristic feature for wild-type GFF cells.

Our initial experiments based on the design of the vector construction targeted exon 0 locus of goat endogenous K-ras were failed. Based on the genome data [24], [25], goat K-ras promoter region is very rich in G and C, lacks TATA and CCAAT boxes, and contains sequence similarities with other house-keeping genes, such as dihydrofolate reductase (DHFR) and epidermal growth factor (EGF) receptor genes. The promoter of the c-K-ras gene consists of multiple elements, and initiation of transcription occurs at multiple sites. Sequences overlapping with the 5′ end untranslated exon; therefore, downstream from the major transcription initiation sites is important (although not sufficient) for transcription. Considering that any mismatch induced by homologous recombination of exon 0 would greatly impair the promoter's activity of the upstream elements, we changed our design to target exon 1.

Among 56 drug-resistant colonies of GFFs, we were able to obtain two colonies with the right homologous recombination, i.e., with a target enrichment efficiency (TRE) of 3%. Compared to the efficiencies of 1–2% [26], [27] in murine embryonic stem cells, our TRE is relatively high but similar to other reports for homologous recombination in sheep, goats, and cattle [28]. The use of a promoter-less gene-targeting vector could further enhance the target efficiency.

Based on the structure of the vector, the ratio of expression level between the endogenous goat *K-ras* and mutated hK-ras would be 1∶1. The observation of cell transformation indicated that the mutated hK-ras gene product replaced the function of endogenous goat K-ras gene and remained in an active GTP-bound state; thus, contributed to the uncontrolled cellular proliferation for tumor formation. K-ras is a highly conservative gene, and a blast analysis from NCBI revealed 95% sequence similarity between human K-ras and goat K-ras. Since the mutated region in K-ras is identical in mouse, goat, and human, we expected that the outcome would be similar if we mutated the goat K-ras gene.

It is also interesting to notice that a single mutation of K-ras can initiate the transformation of a fibroblast cell into a tumorigenic cell without the need of a co-factor. Similar results were observed in murine and human cell lines [29], [30]. Although it is widely accepted that fibroblasts play a prominent role in the progression, growth, and spread of cancers, their functions are normally achieved through microenvironment niches, where their production of growth factors, chemokines, and extracellular matrix facilitates the angiogenic recruitment of endothelial cells and pericytes. The transformation of fibroblast to tumor cells via hK-ras mutation reported herein supports the discussions of expanded role of fibroblast in cancer as direct tumor genesis instead of the current view of its function as an influence factor [31]–[33].

As the first experiment to generate xenograft mice using goat cancer cells, the tumor growth in the nude mice was relatively slow compared to that using human cancer cells (Figure 5). This could be due to the nature of goat cells and the presence of normal endogenous K-ras (50% theoretically) in addition to the mutated human K-ras gene in the transgenic cells.

The integration of a nuclear imaging reporter gene (HSV1-tk in this case) enables imaging and quantifying mutated K-ras gene product *in vivo* as well as the subsequent phenotypes. Such capability is very important to facilitate generating a large-animal model of cancer. Rather than relying purely on the appearance of disease phenotype, which may take a much longer time in large animals (or may not happen at all), one could follow the stages of model development by assessing the oncogene product levels *in vivo* and adjusting the strategies and approaches at an early stage based on the imaging results. In addition, the development of cloned transgenic large animals and the ability to evaluate pharmacologic parameters through imaging would significantly lower the expense and the numbers of animals needed for any pharmacologic studies.

Using the transgenic cells as donor cells, five K-ras transgenic goat kids have been produced at USU using the somatic cell nuclear transfer technique. Tissue specific activation of K-ras and HSV1-tk genes will be carried out once the goats are 6 months to a year old using Cre recombinant adenovirus. Depending on the delivery site of Cre recombinant adenovirus, the transgenic goats could be potential models for many types of cancer models, ranging from lung cancer to kidney cancer.

## Supporting Information

File S1 **Contains the files Table S1 and Figure S1-S7.** Table S1. Primer sequences for constructing target vector pKO2.1-LSL-hK-ras^G12D^-IRES-HSV1-tk. Figure S1. pLOX-hKras^G12D^-iresTK was verified by the digestion analysis of restriction endonuclease. Lane1-5: 6 colonies of pLOX-hKras^G12D^-iresTK. Lane 6: pLOX-gfp-iresTK. Figure S2. pKO2.1-long arm was verified by the digestion analysis of restriction endonuclease. Lane 1: plasmid pKO2.1-long arm; Lane 2: pKO2.1-long arm/AgeI; Lane 3: pKO2.1-long arm/BglII; Lane 4: pKO2.1-long arm/AflII; Lane 5: pKO2.1-long arm/NotI. Figure S3. Agarose gel electrophoresis of inserted PCR fragments and linearized vector. Lane 1: splicing acceptor (400 bp); Lane 2: K-ras^G12D^-iresTK (2 kb); Lane 3: SA-K-ras^G12D^-iresTK (2.4 kb); Lane 4: LoxP-neomycin-stop-LoxP (2.7 kb); Lane 5: 5′-homologous arm (2 kb); Lane 6: 3′-homologous arm (7 kb); Lane7: linearized pKO2.1-long arm (10 kb). Figure S4. Agarose gel electrophoresis of In-fusion cloning reaction. Lane 1: linearized vector (10 kb); Lane 2: 5′-homologous arm (2 kb); Lane 3: LSL (2.7 kb); Lane 4: SA-K-ras^G12D^-iresTK (2.4 kb); Lane 5: linearized recombinant vector (17 kb). Figure S5. Identification of pko2.1-LSK-K-ras^G12D^-iresTK vector digested with restriction enzymes and confirmation of three positive colonies. Figure S6. Depicting the design of screening primers. V01 and V02, V01 and V03 as the screening primer pairs could detect the homologous recombination taken place in the short arm. V04 and V05 as the screening primer pair could detect the homologous recombination taken place in the long arm. Figure S7. Depicting primer design for confirming the excision of LoxP embedded neo gene. V06 and V07 were designed outside of the two LoxP sites and would lead to amplifying different length of fragments from two different alleles.
(ZIP)
