# Supplementary material for: Activating the Expression of Human K-rasG12D Stimulates Oncogenic Transformation in Transgenic Goat Fetal Fibroblast Cells
Source: PLoS One. 2014 Mar 4;9(3):e90059. doi: 10.1371/journal.pone.0090059 (PMC3942380; doi:10.1371/journal.pone.0090059)
Supplement: File S1 — Contains the files Table S1 and Figure S1-S7. Table S1. Primer sequences for constructing target vector pKO2.1-LSL-hK-rasG12D-IRES-HSV1-tk. Figure S1. pLOX-hKrasG12D-iresTK was verified by the digestion analysis of restriction endonuclease. Lane1-5: 6 colonies of pLOX-hKrasG12D-iresTK. Lane 6: pLOX-gfp-iresTK. Figure S2. pKO2.1-long arm was verified by the digestion analysis of restriction endonuclease. Lane 1: plasmid pKO2.1-long arm; Lane 2: pKO2.1-long arm/AgeI; Lane 3: pKO2.1-long arm/BglII; Lane 4: pKO2.1-long arm/AflII; Lane 5: pKO2.1-long arm/NotI. Figure S3. Agarose gel electrophoresis of inserted PCR fragments and linearized vector. Lane 1: splicing acceptor (400 bp); Lane 2: K-rasG12D-iresTK (2 kb); Lane 3: SA-K-rasG12D-iresTK (2.4 kb); Lane 4: LoxP-neomycin-stop-LoxP (2.7 kb); Lane 5: 5′-homologous arm (2 kb); Lane 6: 3′-homologous arm (7 kb); Lane7: linearized pKO2.1-long arm (10 kb). Figure S4. Agarose gel electrophoresis of In-fusion cloning reaction. Lane 1: linearized vector (10 kb); Lane 2: 5′-homologous arm (2 kb); Lane 3: LSL (2.7 kb); Lane 4: SA-K-rasG12D-iresTK (2.4 kb); Lane 5: linearized recombinant vector (17 kb). Figure S5. Identification of pko2.1-LSK-K-rasG12D-iresTK vector digested with restriction enzymes and confirmation of three positive colonies. Figure S6. Depicting the design of screening primers. V01 and V02, V01 and V03 as the screening primer pairs could detect the homologous recombination taken place in the short arm. V04 and V05 as the screening primer pair could detect the homologous recombination taken place in the long arm. Figure S7. Depicting primer design for confirming the excision of LoxP embedded neo gene. V06 and V07 were designed outside of the two LoxP sites and would lead to amplifying different length of fragments from two different alleles. (ZIP) [file pone.0090059.s001.zip › Supplementary/Supplementary-final.docx]

**Supplementary Information**

Construction of recombinant target vector pKO2.1-LSL-hK-ras^G12D^-IRES-HSV1-tk

The recombinant target vector was constructed using in-fusion cloning technology in 4 steps.

Step1: Constructing pLOX-hKras^G12D^-iresTK vector. hK-ras^G12D^ was amplified from pcDNA3-*KRAS*^G12D^ and pLOX-gfp-iresTK vector was double digested by XohI/BamHI After removing gfp, the fragment of hK-ras^G12D^ was cloned to form pLOX-hK-ras^G12D^-iresTK vector, which was structurally confirmed by the digestion analysis of restriction endonuclease (Fig.S1) as well as verified by sequencing.

Step2: Subcloning 3’-homologous arm into pKO2.1. The 7 kb homologous arm fragment was amplified from goat genome DNA and subcloned into pKO2.1. The recombination plasmid pKO2.1-long arm was identified, which was structurally confirmed by the digestion analysis of restriction endonuclease (Fig.S2).

Step3: Amplifying target fragments. Table S1 described the sequences of PCR primers used for target fragment amplification. The primer design was based on the strategy of adding a 15 base sequence homologous to the 15 bases at one end of the DNA fragment to which it will be joined to the 5'-end to facilitate In-Fusion Cloning. The DNA fragments of 5'-homolous arm and splicing acceptor sequence (SA) were amplified from goat genome DNA. The fragments of loxP-Stop-LoxP (LSL) were constructed from PGKneotpAlox2 and the fragment of hK-ras^G12D^-iresTK was amplified from pLOX-hKras^G12D^-iresTK. Fragments of SA and hK-ras^G12D^-iresTK were cloned into T-vector and SA-hK-ras^G12D^-iresTK as a whole fragment was amplified. Fig.S3 showed all the amplified fragments and inserted vector.

Step4: Cloning target vector by In-fusion PCR. The linearized vector of Pko2.1-long arm was generated using unique restriction enzymes-AgeI. In-fusion cloning reaction with a total 10 μL volume was carried out, containing 3 fragments obtained from Step 3 and the linearized vector with a molar ratio of 2:2:2:1. Fig. S4 confirmed the sizes of four fragments and the whole target plasmid. After transformation, 29 colonies were screened by colony PCR and 3 positive colonies were identified and the corresponding plasmids were verified by the digestion analysis of restriction endonuclease (Fig.S5). The junction sequences among the fragments were verified by sequencing.
