# Supplementary material for: Activating the Expression of Human K-rasG12D Stimulates Oncogenic Transformation in Transgenic Goat Fetal Fibroblast Cells
Source: PLoS One. 2014 Mar 4;9(3):e90059. doi: 10.1371/journal.pone.0090059 (PMC3942380; doi:10.1371/journal.pone.0090059)
Supplement: File S1 — Contains the files Table S1 and Figure S1-S7. Table S1. Primer sequences for constructing target vector pKO2.1-LSL-hK-rasG12D-IRES-HSV1-tk. Figure S1. pLOX-hKrasG12D-iresTK was verified by the digestion analysis of restriction endonuclease. Lane1-5: 6 colonies of pLOX-hKrasG12D-iresTK. Lane 6: pLOX-gfp-iresTK. Figure S2. pKO2.1-long arm was verified by the digestion analysis of restriction endonuclease. Lane 1: plasmid pKO2.1-long arm; Lane 2: pKO2.1-long arm/AgeI; Lane 3: pKO2.1-long arm/BglII; Lane 4: pKO2.1-long arm/AflII; Lane 5: pKO2.1-long arm/NotI. Figure S3. Agarose gel electrophoresis of inserted PCR fragments and linearized vector. Lane 1: splicing acceptor (400 bp); Lane 2: K-rasG12D-iresTK (2 kb); Lane 3: SA-K-rasG12D-iresTK (2.4 kb); Lane 4: LoxP-neomycin-stop-LoxP (2.7 kb); Lane 5: 5′-homologous arm (2 kb); Lane 6: 3′-homologous arm (7 kb); Lane7: linearized pKO2.1-long arm (10 kb). Figure S4. Agarose gel electrophoresis of In-fusion cloning reaction. Lane 1: linearized vector (10 kb); Lane 2: 5′-homologous arm (2 kb); Lane 3: LSL (2.7 kb); Lane 4: SA-K-rasG12D-iresTK (2.4 kb); Lane 5: linearized recombinant vector (17 kb). Figure S5. Identification of pko2.1-LSK-K-rasG12D-iresTK vector digested with restriction enzymes and confirmation of three positive colonies. Figure S6. Depicting the design of screening primers. V01 and V02, V01 and V03 as the screening primer pairs could detect the homologous recombination taken place in the short arm. V04 and V05 as the screening primer pair could detect the homologous recombination taken place in the long arm. Figure S7. Depicting primer design for confirming the excision of LoxP embedded neo gene. V06 and V07 were designed outside of the two LoxP sites and would lead to amplifying different length of fragments from two different alleles. (ZIP) [file pone.0090059.s001.zip › Supplementary/table-s1.docx]

**Table S1.**

| **Primer** | **Fragment** | **Template** | **Sequence of primer (5’-3’)** |
| --- | --- | --- | --- |
| P1 | 5’-arm | Goat genome DNA | TCTAGAGCGGACCGGTGCTGACTCGAACGTGTAGTGGACTA |
| P2 | 5’-arm | Goat genome DNA | TCTAGAGCCTTAAGCGGTTGACAGCTACCACCACAGTATTC |
| P3 | LSL | PGKneotpAlox2 | CGCTTAAGGCTCTAGAACTAGTGGATCCGGA |
| P4 | LSL | PGKneotpAlox2 | CCCATATG ATCGATAAGCTAGCTTGGGCTGC |
| Psa(F) | SA | Goat genome DNA | TATCGATCATATG GGAGAGCAGCGCACTCAACACTT |
| Psa(R) | SA | Goat genome DNA | CAGTCATTTTCAGCAGGCCTTAT |
| P5 | Kras-TK | pLOX-hKrasG12D-iresTK | ATAAGGCCTGCTGAAAATGACTGAATATAAACTTGTGGTAG |
| P6 | Kras-TK | pLOX-hKrasG12D-iresTK | ATCCACAAAGACCGGTACGTTATACAGGTCGCCGTT |
| P7 | 3’-arm | Goat genome DNA | GCACCGGTCTTTGTGGATGAATATGATCCTACG |
| P8 | 3’-arm | Goat genome DNA | ATTTGCGGCCGCGCAAAGTGAACCTGGGATATACTGT |

The sequence of original primers is shown in black and vector-specific part is in red. The underlined represented the restriction enzyme.
